# Fabry Disease Cardiomyopathy: A Review of the Role of Cardiac Imaging from Diagnosis to Treatment

**DOI:** 10.31083/j.rcm2306192

**Published:** 2022-05-27

**Authors:** Laura Fuertes Kenneally, María Isabel García-Álvarez, Eloísa Feliu Rey, Ana García Barrios, Vicente Climent-Payá

**Affiliations:** ^1^Heart Failure and Inherited Cardiac Diseases Unit, Cardiology Department, Hospital General Universitario Dr. Balmis, Alicante Institute for Health and Biomedical Research (ISABIAL), 03010 Alicante, Spain; ^2^Radiology Department, Hospital General Universitario Dr. Balmis, Alicante Institute for Health and Biomedical Research (ISABIAL), 03010 Alicante, Spain

**Keywords:** Fabry disease, multimodal imaging, echocardiography, cardiac magnetic resonance imaging, left ventricular hypertrophy, inflammation and fibrosis

## Abstract

Fabry disease is a rare X-linked inherited lysosomal storage disorder caused by the absence or reduction of alfa-galactosidase A activity in lysosomes, resulting in accumulation of glycosphingolipids in various tissues. The main organ affected is the heart, which frequently manifests as left ventricular hypertrophy and can ultimately lead to cardiac fibrosis, heart failure, valve disease, cardiac conduction abnormalities and sudden cardiac death. Today we know that myocyte damage starts before these signs and symptoms are detectable on routine studies, during the designated pre-clinical phase of Fabry disease. The initiation of specific therapy for Fabry disease during the early stages of the disease has a great impact on the prognosis of these patients avoiding progression to irreversible fibrosis and preventing cardiovascular complications. Cardiac imaging has become an essential tool in the management of Fabry disease as it can help physicians suspect the disorder, diagnose patients in the early stages and improve outcomes. The recent development of novel imaging techniques makes necessary an update on the subject. This review discusses the role of multimodal imaging in the diagnosis, staging, patient selection for treatment and prognosis of Fabry disease and discusses recent advances in imaging techniques that provide new insights into the pathogenesis of the disorder and the possibility of novel treatment targets.

## 1. Introduction 

Fabry disease (FD) is a rare X-linked inherited lysosomal storage disorder caused by the absence or reduction of alfa-galactosidase A activity (α-Gal A) in lysosomes. More than 900 mutations of the α*-Gal A* gene have been identified to date [1] and the reported incidence is between 1 in 40,000 and 1 in 117,000 male births. This figure may be underestimated as recent screening suggests a prevalence of up to 1 in 8800 newborns with the inclusion of late-onset and milder GLA variants [2].

The enzyme deficiency results in the accumulation of globotriaosylceramide (Gb3) and its derivative, globotriaosylsphingosine (lyso-Gb3), in various organs including the heart, kidneys, gastrointestinal tract, vasculature and peripheral nervous system. The heart is the most frequently affected organ with more than 50% of all FD patients having cardiac involvement [3] and represents the main cause of impaired quality of life and death in these patients [4, 5]. Furthermore, the heart can be the only organ affected in men with specific gene mutations and in women carriers suffering from the so-called “cardiac Fabry variant” [6]. All cardiac structures can be affected in FD including the myocardium, conduction system and valves, giving rise to multiple manifestations that include left ventricular hypertrophy (LVH), arrhythmias, myocardial fibrosis and functional impairment.

Due to the availability of specific treatment for FD such as enzyme replacement therapy (ERT) [7, 8] and the pharmacological chaperone Migalastat [9], early diagnosis has become essential to slow the progression of the disease, improve prognosis and avoid the development of irreversible fibrosis. Evidence suggests that the best outcomes occur with early initiation of treatment [10]. In this regard, cardiac imaging is key to establish a correct diagnosis because it can identify “red flags” that raise the suspicion of this rare disorder, can rule out other causes of LVH and help detect the disease as early as possible via subclinical abnormalities. Recent discoveries and the development of cutting-edge imaging techniques have also shed light on the underlying mechanisms of FD and aided disease staging with important clinical implications for the correct selection of candidates for treatment. Lastly, we cannot undermine the prognostic value this provides.

The aim of this article is to raise awareness of the existence of this rare disorder and review the role of multimodal imaging in the diagnosis, staging, patient selection for treatment and prognosis of FD.

## 2. Diagnosis and Early Detection 

Diagnosis of FD is often delayed due to the rarity of the condition, the lack of awareness among clinicians and the diversity and non-specificity of presenting symptoms. Data from the Fabry Outcome Survey (FOS), showed that patients with FD were diagnosed 13.7 years after the onset of symptoms in males and 16.3 years in females, with a maximum delay of >50 years for some patients [11] despite the novel advancements in diagnosis and screening techniques, the diagnostic delay has not improved in recent years [12].

**Fig. 1.**
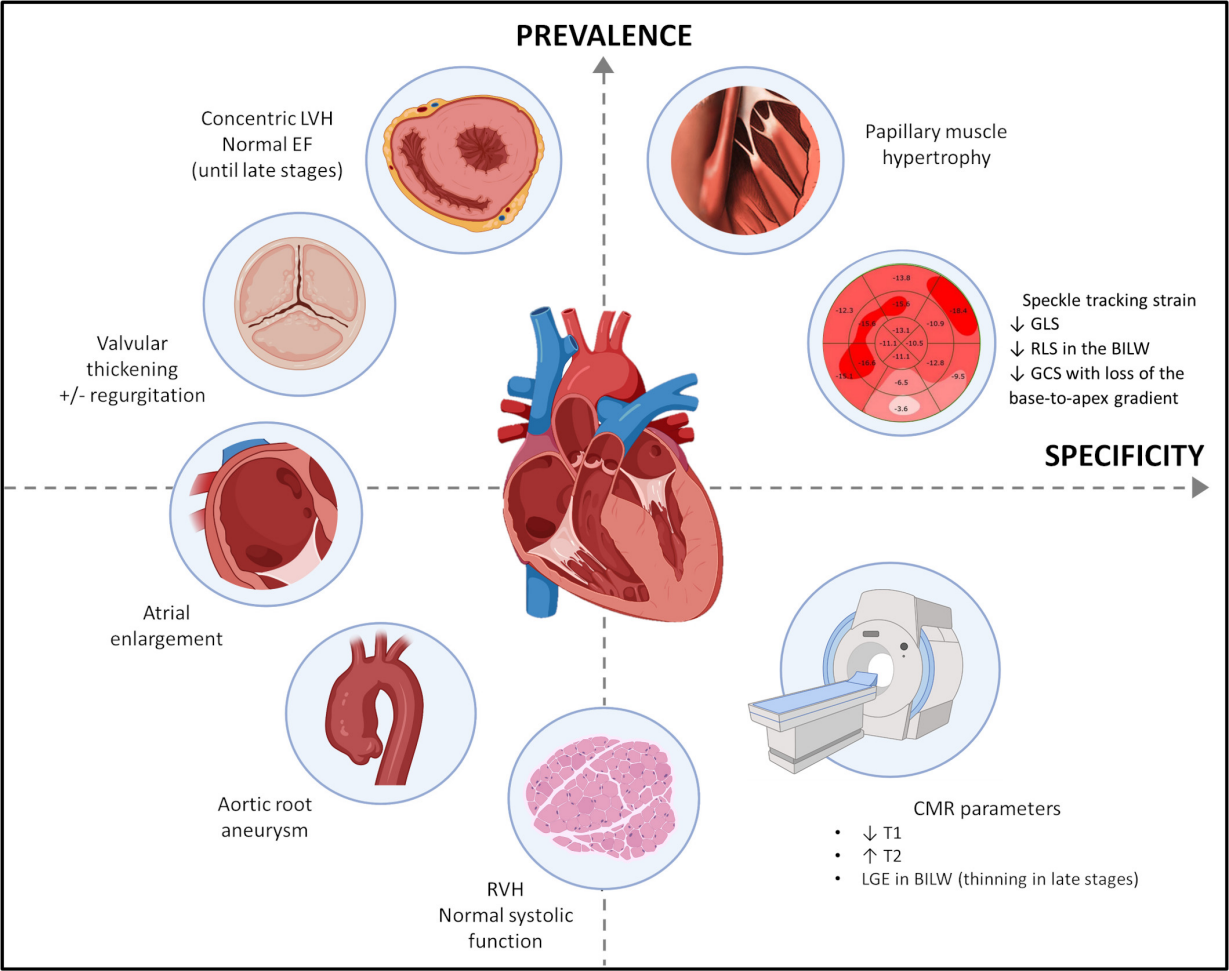
**Imaging findings and “red flags” of Fabry disease cardiomyopathy along with their relative prevalence and specificity**. CMR, cardiac magnetic resonance; BILW, basal inferolateral wall; EF, ejection fraction; GCS, global circumferential strain; GLS, global longitudinal strain; LGE, late gadolinium enhancement; LVH, left ventricular hypertrophy; RLS, regional longitudinal strain; RVH, right ventricular hypertrophy.

The echocardiography is considered the first-line test to detect cardiac involvement in FD patients because it is widely availability, low cost and noninvasive. Fig. 1 summarizes common imaging features in FD. However, we would like to emphasize that none of the following findings are pathognomonic.

### 2.1 Cardiac Structure

The hallmark feature of FD cardiomyopathy is LVH which is detected in up to 50% of patients [13]. Conversely, the prevalence of FD in patients with unexplained LVH varies widely from 0–12% in previous studies due to different inclusion criteria and study design [14, 15, 16]. A recent re-analysis of 5491 patients with an initial diagnosis of LVH and/or HCM reported a prevalence of FD of 0.94% in males and 0.90% in females [17].

LVH is more prevalent and has an earlier onset in men compared to women (42.0 ± 4.5 vs 50.1 ± 12.0 years, respectively) [3]. Different levels of residual α-Gal A activity between male and female patients could account for these findings. A large multinational cohort of FD patients observed that a lower α-Gal A activity correlated with greater LV wall thickness [18]. As FD is inherited in an X-linked pattern, male patients with a mutation in the *GLA* gene usually have lower residual enzymatic activity and more severe manifestations than female patients that have two copies of the gene and therefore display a broader spectrum of disease severity.

**Fig. 2.**
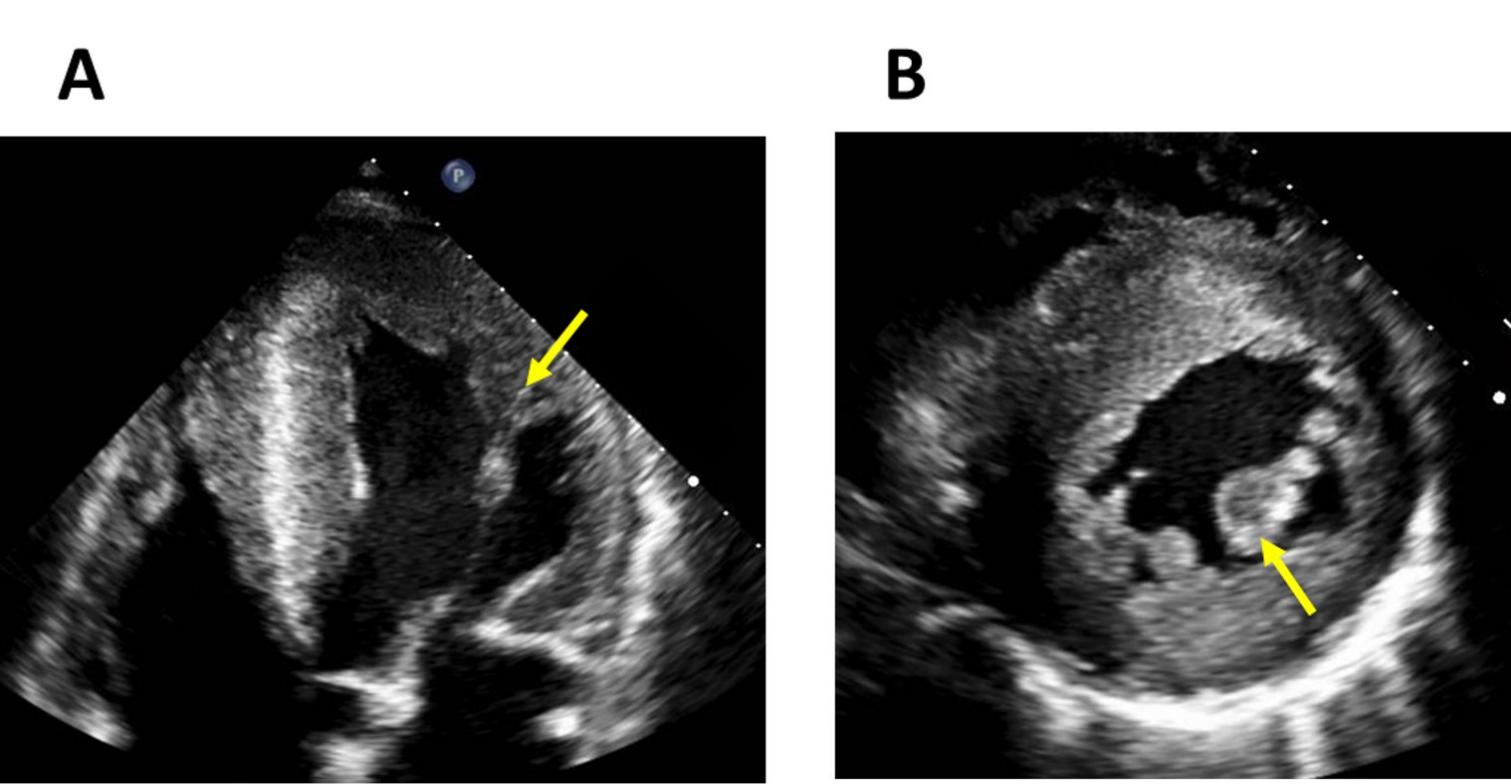
**Left ventricular and papillary muscle hypertrophy in Fabry disease**. Echocardiography four-chamber view (A) and short-axis CMR image (B) that shows a severe left ventricular hypertrophy and papillary muscle hypertrophy (arrows) in a 47-year-old male patient with Fabry disease.

**Fig. 3.**
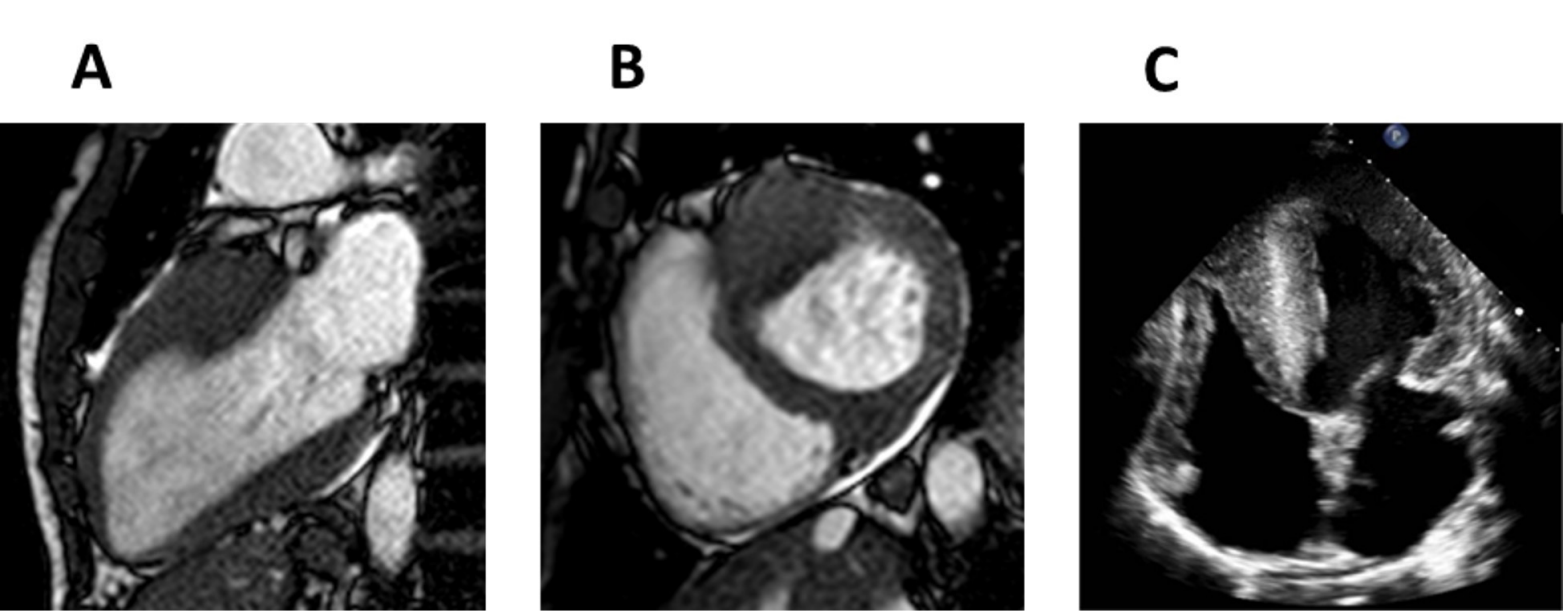
**Asymmetrical septal hypertrophy mimicking hypertrophic cardiomyopathy in a patient with Fabry disease**. Long-axis (A) and short-axis (B) CMR images and an echocardiography apical four-chamber view (C) of a patient with Fabry disease showing an asymmetrical septal hypertrophy that mimics the pattern seen in hypertrophic cardiomyopathy.

LVH in FD typically presents a concentric pattern (Fig. 2) without resting left ventricular outflow tract obstruction (LVOTO). However, obstructive forms, asymmetric septal (Fig. 3), apical and eccentric hypertrophy have also been described [19, 20]. In fact, LVOTO may be more prevalent and have a greater impact on symptoms than was previously thought. In a small cohort of 14 patients, LVOTO was revealed by exercise stress echocardiography in six patients with refractory symptoms [21]. A smaller cavity size and papillary muscle (PM) hypertrophy were speculated to be involved in the LVOTO.

Disproportionate hypertrophy of PM could also be a useful marker of FD (Fig. 2). Niemann *et al*. [22] showed that PM area (measured by echocardiography in the mid-ventricular short axis view) and the ratio of PM area to the circumference of the left ventricle (LV) were significantly higher in FD patients compared to patients with other diseases that cause LVH (amyloidosis, aortic stenosis, hypertrophic cardiomyopathy (HCM), etc.) with a cut-off value of 3.6 ${\mathrm{cm}}^{2}$ and 0.18 respectively. The combination of both parameters yielded a sensitivity of 75% and specificity of 86% for diagnosing FD in patients with LVH. Furthermore, abnormalities in PM structure and function have been proposed as a mechanism of mitral regurgitation in these patients. Nonetheless, the presence of hypertrophic PM alone does not suffice to differentiate FD from other etiologies of LVH [23].

In 2006, Pieroni *et al*. [24] suggested that the binary sign was a hallmark feature of FD as it occurred in up to 83% of patients that participated in the study. The binary sign refers to the appearance of the LV endocardial border on echocardiography; a hyperechogenic region in the endocardial surface adjacent to a relatively low echo intensity layer in the subendocardial region creates a clear black and white interface. However, further studies have detected a lower prevalence of the sign, only 29% of patient is one prospective study [25]. The sensitivity and specificity in the same study was 28% and 80% respectively [25].

Right ventricular hypertrophy (RVH) can also be found in FD patients with a prevalence that varies between 31% and 71% and increases with age [26, 27]. The extent of the hypertrophy correlates with the degree of LVH and the stage of the disease [26]. However, right ventricular (RV) systolic dysfunction as measured by tricuspid annular plane systolic excursion is rare, even in the presence of severe RVH, and when present is associated to advanced stages of the disease [28]. Despite normal systolic and diastolic function, patients may exhibit subclinical RV systolic impairment on speckle-tracking strain imaging [29]. Unlike LVH, RVH appears to affect males and females alike and systolic function and the degree of hypertrophy has not been found to influence prognosis [30]. This differs from patients suffering from amyloidosis or HCM, in whom RVH and systolic function were associated with worse outcomes [31, 32]. The presence of fibrosis in the RV also seems to be less common than in the LV. In a cohort of 75 patients with FD [27], none of them presented late gadolinium enhancement (LGE) in the RV free wall on cardiac magnetic resonance imaging (CMR), not even those with severe LV replacement fibrosis. The authors speculated that differences in RV and LV geometry and wall stress might be a possible explanation. However, these finding were not confirmed by histological analysis due to ethical reasons*. *A histological examination of the heart of three patients with FD found replacement and interstitial fibrosis in both the left and right ventricle, although it was more extensive in the LV (17% vs 9% respectively) [33]. The RV also shows a different response to treatment compared to the LV. Niemann* et al*. [27] observed that ERT does not improve RV morphological or functional parameters during a 2.3-year follow-up, raising questions about the underlying pathological mechanism of RV involvement in FD. Although biopsy studies show accumulation of Gb3 in both ventricles [33] it could be that the development of RVH is more related to trophic factors than to direct storage of Gb3 [34]. Unlike other causes of RVH, increased afterload or ventricular interdependence have not been demonstrated to play a major role in the development of RVH in FD patients [30].

Other echocardiographic findings in FD may include: left atrial enlargement and dysfunction, aortic or mitral valve thickening with or without mild to moderate regurgitation and LV hyper-trabeculation and non-compaction [35]. Aortic dilation has also been reported with a special predilection for males and advanced stages of the disease. In the largest study to date investigating aortic remodeling in FD patients, aortic dilation at the sinus of Valsalva and ascending aorta was identified in 32.7% and 29.6% of males, respectively [36]. Aortic aneurysms were less prevalent (9.6% of male patients) [36]. Their clinical significance with respect to the risk of dissection, rupture or need for surgery remains uncertain. Lastly, thinning of the basal inferolateral wall (BILW) of the LV is infrequent and has been associated with worsening functional capacity and cardiac death [37].

### 2.2 Cardiac Function

Left ventricular ejection fraction (LVEF) in FD patients is usually preserved until late stages of the disease. However, LVEF has shown to have a low sensitivity to detect myocardial dysfunction [38].

In recent years, novel techniques have been developed such as Speckle-tracking or Tissue Doppler Imaging (TDI) that are able to detect systolic or diastolic dysfunction in earlier stages (even when LVEF is normal) helping to diagnose subclinical cardiomyopathy. In the following sections we will revise the utility of each of these techniques separately.

#### 2.2.1 Tissue Doppler Imaging

TDI uses Doppler ultrasound imaging to detect frequency shifts of ultrasound waves reflected from the myocardium to calculate myocardial velocity [39]. This technique has demonstrated to be a helpful screening tool for preclinical cardiac damage in FD [10, 40]. A study by Perioni *et al*. [41] compared TDI velocities in three groups: patients with FD and LVH, patients with FD without LVH and healthy volunteers. They concluded that patients with FD had decreased systolic and diastolic TDI velocities (e’, a’ and S’) and elevated E/e’ compared to normal controls. This was true for both patients with and without LVH, although TDI dysfunction was more pronounced when LVH was present. These results are similar to the ones found in a study carried out by our group which included 50 FD patients. Our study showed that patients with FD had lower systolic and diastolic TDI velocities than healthy volunteers [42]. Similar to the results of Pieroni *et al*. [41], we have found that FD patients without LVH showed a tendency to a higher E/e’ ratio when compared to the control group but no statistically significant differences were found [42].

The isovolumetric contraction time has also proven to be useful as it was identified as the best parameter for detecting preclinical cardiomyopathy in FD patients with a sensitivity of 100% and specificity of 91%, considering a cut off value of <105 msec [43]. Lastly, TDI can be used to detect decreased left atrial compliance in patients with FD [44].

#### 2.2.2 Two-Dimensional Speckle-Tracking

Speckle-tracking is a novel technique that tracks frame-to-frame movements of acoustic markers or “speckles” on the myocardium, allowing the assessment of myocardial strain. Myocardial strain is an intrinsic mechanical property of the myocardium that measures the deformation of the cardiac wall that is, the fractional change in the length of a myocardial segment. The change of strain per unit of time is referred to as strain rate (SR) [45]. Speckle-tracking offers additional advantages over TDI such as the non-dependence of the measurement angle and therefore the ability to assess regional function of all myocardial segments in two dimensions. It also possesses a greater reproducibility [46].

**Fig. 4.**
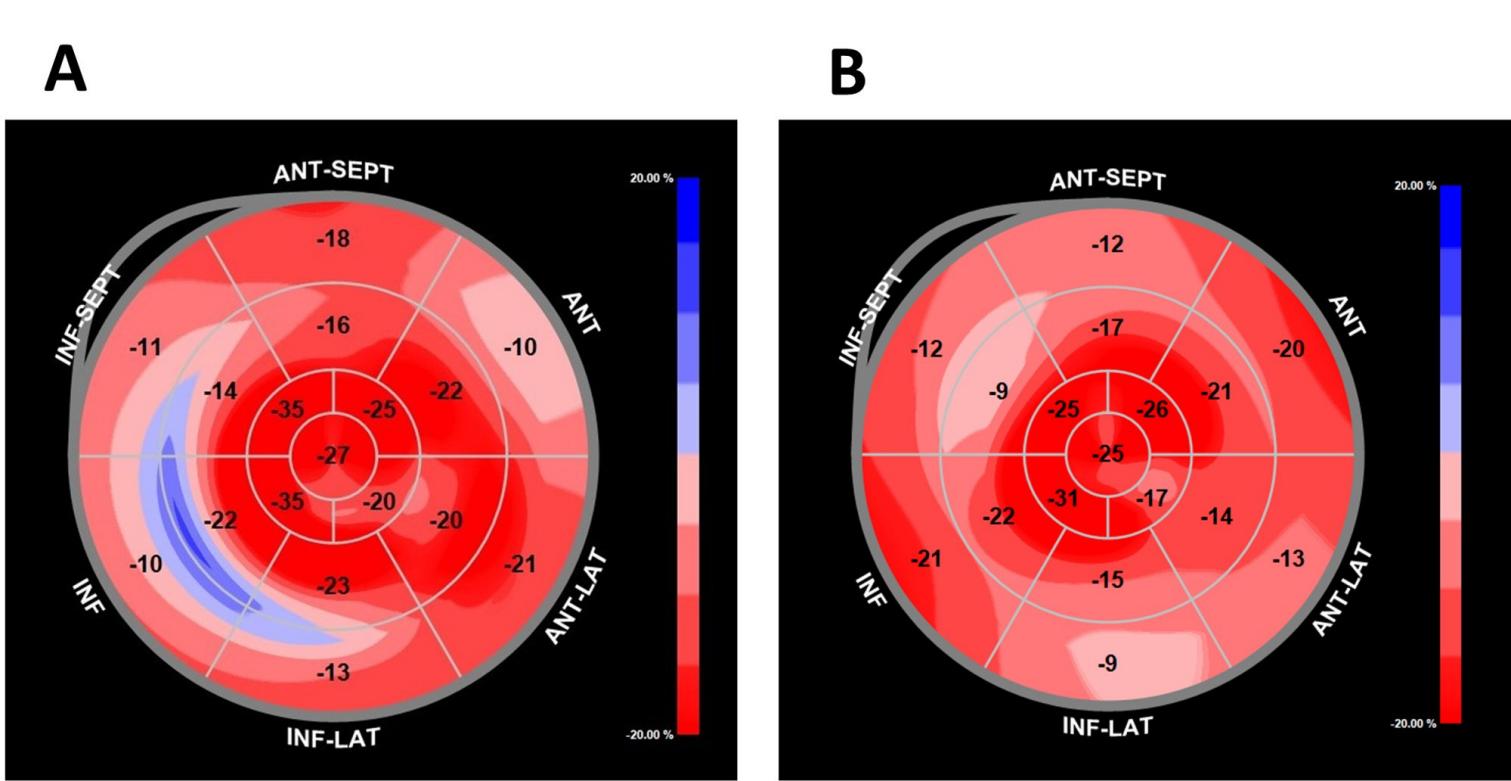
**Speckle-tracking strain imaging in Fabry disease**. Speckle-tracking image of a patient with Fabry disease that shows a reduction in the regional strain of the basal inferolateral wall, the most frequently affected segment in Fabry disease (A). Speckle-tracking image that shows a decreased strain value in the basal and mid segments of the left ventricle with an “apical sparing pattern” that can be found in some patients (B).

Measuring myocardial strain can help detect early functional impairment in FD patients. A reduction in global longitudinal strain (LS) precedes the deterioration of LVEF and the development of LVH and cardiac symptoms [47, 48]. This reduction in global LS in the early stages is usually due to a regional decrease in LS in the BILW [41, 49]. These findings coincide with the results of our own research [42] that showed that FD patients had a lower global LS (–20.0% vs –22.0%; *p* = 0.024) compared to normal controls. The BILW was also the most affected segment and showed the greatest differences regarding healthy subjects (Fig. 4).

Subsequent studies showed a more global alteration of LS in basal and mid-LV segments compared to healthy controls contributing to an “apical sparing pattern” that can also be observed in cardiac amyloidosis [50].

A recent study performed multilayer strain images in newly diagnosed FD patients and compared them to healthy controls. They found that all myocardial layers had lower strain values in FD patients, but reduction of subepicardial LS was the most significant and best discriminated FD patients from normal controls [51]. Accordingly, FD patients had a higher strain gradient (subendocardial LS – subepicardial LS). This finding was evident even in patients without LVH indicating that damage to subepicardial fibers is present in the initial stages of the disease.

The LV was not the only cardiac chamber to have impaired function when assessed by speckle-tracking. Reduced RV global and free wall systolic strain has also been described in the literature [29] and in our study we found that FD patients had a lower global left atrial strain compared to healthy individuals (31.9% vs 56.1%; *p *< 0.001). Global left atrial strain was inversely correlated with LV wall thickness (r = –0.565; *p *< 0.001) [42].

Speckle-tracking could also help distinguish the condition from other causes of LVH. Patient with FD have a reduction in global circumferential strain (CS) with a loss of the normal base-to-apex gradient [52]. On the contrary, HCM patients had higher global CS and preserved the base-to-apex gradient. Thus, this pattern of deformation is thought to be specific to FD cardiomyopathy and could be caused by the greater impairment of subepicardial fibers which are mainly responsible for global CS, while global LS is largely attributed to subendocardial fibers [53].

In summary, longitudinal, circumferential and radial strain are reduced in FD patients, whereas the basal segments, especially the BILW and the subepicardial layers where the most affected and the earliest to occur. Hence, strain analysis of the BILW or subepicardial segment could be used to screen for cardiac involvement in FD patients without LVH.

#### 2.2.3 Strain Rate Imaging

Regarding strain rate (SR) imaging, patients with FD have reduced radial and longitudinal SR and peak systolic SR [54]. The mentioned deterioration in LV function measured by SR imaging seems to follow a specific order as the disease progresses with potential implications for staging. Weidemann *et al*. [55] demonstrated that LV longitudinal SR was impaired earlier than radial function and started in the BILW. Patients in later stages developed impaired radial SR and worsening longitudinal SR in the septal wall.

## 3. Differential Diagnosis

**Fig. 5.**
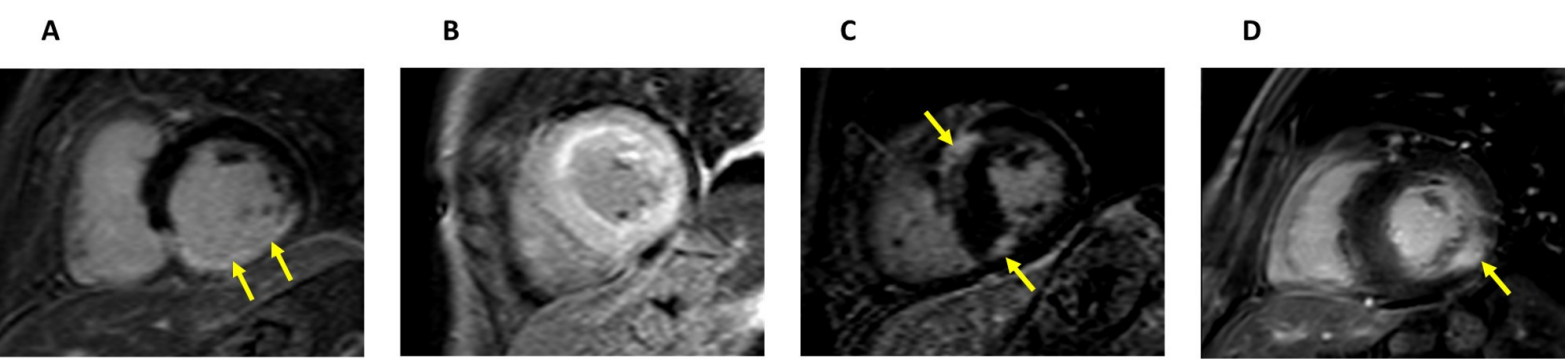
**Different patterns of late gadolinium enhancement in CMR images of diseases that cause left ventricular hypertrophy**. (A) Short-axis late gadolinium enhancement (LGE) CMR image of a patient that suffered an inferior myocardial infarction that shows transmural LGE in the inferior wall (arrows). (B) Short-axis LGE CMR image of a patient with amyloidosis, demonstrating a diffuse circumferential pattern of LGE. (C) Short-axis LGE CMR image of a patient with hypertrophic cardiomyopathy and LGE at the junctions of the ventricular septum and right ventricle (arrows). (D) Short-axis LGE CMR image of a patient with FD. Note the enhancement in the inferolateral region (arrow).

Differential diagnosis of FD must include other causes of LVH such as chronic afterload increase (hypertension and aortic stenosis), HCM and infiltrative cardiomyopathies (cardiac amyloidosis, Friedreich’s ataxia and Danon disease). Table 1 (Ref. [56, 57, 58, 59, 60, 61, 62, 63]) and Fig. 5 summarize the differential diagnosis of FD.

**Table 1.** **Differential diagnosis of Fabry disease with other causes of left ventricular hypertrophy**.

	FD	HCM	Amyloidosis	Aortic stenosis	Hypertensive heart disease	Danon disease	Friedreich’s ataxia	Mitochondrial myopathy
References	[56, 57, 58]	[56, 57, 58]	[56, 57, 58, 59, 60]	[56, 57, 58]	[56, 57, 58]	[56, 57, 58]	[56, 57, 58, 61, 62]	[56, 57, 58, 63]
Age at presentation	Adulthood	Adolescence/adulthood	Adulthood and elderly (WT-TTR)	Elderly	Adulthood	Early childhood/adolescence	Early childhood/adolescence	Early childhood/adolescence
Inheritance (gene)	X-linked (*GLA* gene)	Mostly AD (≈30–60%); rarely AR, X-linked or maternal	Familial TTR: AD	Non-inherited	Non-inherited	X-linked (*LAMP2* gene)	AR (*FXN* gene)	Mostly maternal
			WT-TTR and AL: non-inherited					
Clinical presentation	Acroparaesthesia, angiokeratoma, impaired sweating, cornea venticillata, renal failure, cerebrovascular disease	Dyspnea, syncope, angina, sudden death	HF, bilateral carpal tunnel, nephrotic syndrome, peripheral neuropathy, hepatomegaly, macroglossia, autonomic dysfunction	Dyspnea, syncope, angina, sudden death	History of hypertension, dyspnea, angina	Typical triad: skeletal myopathy, mental retardation and HCM	Neurological symptoms (dysarthria, loss of reflexes, ataxia, gait abnormality), visual and hearing impairment	Dependent on subtype: mental retardation, sensorineural deafness, muscle weakness, epilepsy, ataxia, ptosis, diabetes mellitus
LVH pattern	Concentric but may also be asymmetric, apical or RV hypertrophy	Mainly asymmetrical septal hypertrophy but may also be concentric or apical	Concentric	Concentric	Concentric with mid LV dilation	Concentric	Concentric	Concentric
			Symmetrical increase in LV and RV hypertrophy			Very thick LV (20–60 mm), RV may or may not be hypertrophic	Increase in LV septal and posterior wall thickness	
Other findings	PM hypertrophy; aortic dilation; valvular thickening and regurgitation	LVOT obstruction; apical aneurysm; mitral apparatus abnormality; SAM; anterior displacement of PM or direct insertion into the MV	Biatrial dilation, valvular thickening, granular appearance of myocardium, restrictive physiology and pericardial effusion	Aortic stenosis	-	-	Granular appearance of myocardium	Dependent on subtype: hypertrophic, dilated, restrictive pattern or non-compaction
Speckle tracking strain	↓ RLS in the BILW	↓ RLS in the area of greatest hypertrophy (septal region)	↓ RLS in the basal and mid regions with apical sparing	↓ GLS (Basal LV segments)	↓ RLS in the hypertrophic area (typically basal septal and most of the basal regions)	-	Nonspecific	-
	↓ GCS Loss of the normal base to apex gradient							
Systolic dysfunction	Decreased EF in advanced stages	Decreased EF in advanced stages	Decreased EF in advanced stages	Rare	Rare	Frequent, and rapid progression	Decreased EF in advanced stages	Frequent and progressive
CMR (Native T1)	↓	Normal	↑	Normal	Normal	Normal	Normal	Dependent on subtype
CMR (LGE pattern)	Midwall of the BILW	Midwall at the junctions of the ventricular septum and RV (patchy)	Subendocardial	Nonspecific	Nonspecific Patchy pattern, predominantly subendocardial	Subendocardial, anterior, lateral, and/or posterior walls with septal sparing	Nonspecific	Midwall of the basal inferolateral wall (in CPEO/KSS)
			Global circumferential					HCM-like LGE (in MELAS)

AD, autosomal dominant; AR, autosomal recessive; AV, atrioventricular; CPEO, chronic progressive external ophthalmoplegia; CMR, cardiac magnetic resonance; CPK, creatine phosphokinase; Gb3, globotriaosylceramide; GCS, global circumferential strain; GLA, galactosidase alpha; ECG, electrocardiogram; ECV, extracellular volume; EF, ejection fraction; FD, Fabry disease; FXN, frataxin; GLS, global longitudinal strain; HCM, hypertrophic cardiomyopathy; KSS, Kearns-Sayre syndrome; LAMP2, Lysosome-associated membrane protein 2; LGE, late gadolinium enhancement; LV, left ventricle; LVH, left ventricular hypertrophy; LVOT, left ventricular outflow tract; Lyso-Gb3, globotriaosylsphingosine; MELAS, mitochondrial encephalomyopathy; MV, mitral valve; NT-proBNP, N-terminal pro-brain natriuretic peptide; PM, papillary muscle; TTR, transthyretin; RLS, regional longitudinal strain; RV, right ventricle; SAM, systolic anterior motion; WPW, Wolff-Parkinson-White; WT-TTR, wild type transthyretin amyloidosis.

## 4. Pathophysiology and Tissue Characterization 

CMR is the gold standard method for measuring ventricular dimensions, wall thickness and LV mass including PM mass [64]. Accordingly, it has become central to early diagnosis and staging of cardiac FD. CMR has the additional advantage of being able to characterize the myocardium by using LGE and magnetic tissue relaxation constants such as T1, T2 and T2* giving us an insight into the following pathological processes: infiltration or storage of sphingolipids (T1), edema or inflammation (T2) and fibrosis (LGE).

### 4.1 T1 Imaging (Storage)

T1 mapping is a CMR imaging technique based on the quantification of the T1 relaxation time of a tissue by using analytical expressions of image-based signal intensities [65]. The T1 relaxation time varies substantially between two tissues. Fibrosis, edema, capillary blood and amyloid increase T1 whereas iron and fat decrease its value [66]. Sarcomeric HCM usually presents a normal T1 value in absence of fibrosis.

Thompson *et al*. [67] reported significantly reduced native T1 values (prior to contrast administration) in FD patients, which is thought to reflect glycosphingolipid storage in the myocardium. T1 in FD was substantially lower when compared to other causes of LVH, highlighting the use of T1 mapping in the differential diagnosis of concentric LVH [68, 69]. Similar to what occurs with speckle-tracking strain and LGE, the degree of native T1 shortening was highest in the inferior and inferoseptal regions [70]. Hence, some authors propose segment-specific T1 cut-off values to better characterize the disease [70].

Reduced T1 values can be detected in up to 59% of FD patients without LVH, indicating that low T1 values are present in early stages [71]. Therefore, T1 mapping has the potential to be used as a screening tool for FD patients. Nonetheless, T1 values do not follow a linear progression with the disease but rather have a biphasic response: lowers with storage and finally increases in advanced disease (pseudo-normalization). Therefore, it fails to predict advanced stages of the disease.

Lastly, T1 mapping allows the non-invasive estimation of myocardial extracellular volume (ECV) by combining T1-times before and after gadolinium administration and the patient’s hematocrit [72, 73]. In contrast to cardiac amyloidosis, ECV in FD patients is similar to healthy subjects as storage predominantly occurs in the intracellular space, except for LGE-positive areas.

### 4.2 T2 Imaging (Inflammation) 

Another CMR imaging technique that is gaining importance in recent years is T2 imaging. T2-weighted sequences make it possible to identify increased water content in tissues which can be inflammatory or noninflammatory (edema) [74].

Previous studies have shown that, when LGE is present, FD patients had elevated T2 values in the LGE segments, particularly in the BILW but also globally. This differed from normal controls and patients with myocardial infarction and was even higher than the T2 elevation seen in HCM.

Aside from its diagnostic value, T2 imaging has provided new insights into the pathophysiology of FD. Classically, FD has been considered simply a storage cardiomyopathy but Gb3 accumulation alone is insufficient to explain the full extent of myocardial abnormalities seen in these patients. Based on T2 imaging studies, Nordin *et al*. [75] hypothesized that FD may in fact be an inflammatory disorder.

The first study to suggest a possible role of inflammation in FD patients was Nappi *et al*. [76]. They pioneered the simultaneous use of positron emission tomography (PET) and CMR to assess cardiac involvement in FD patients. The study showed that patients with LGE and positive T2-weighted short-tau inversion recovery (STIR-T2) sequences also had focal Fluorodeoxyglucose uptake on PET images. Meanwhile, patients with LGE but negative STIR-T2 CMR images did not show focal Fluorodeoxyglucose uptake. Therefore, they were able to differentiate mature fibrosis (or scar tissue) from fibrosis associated to active inflammation.

Subsequently, Nordin *et al*. [77] hypothesized that inflammation could be contributing to the pathogenesis of myocardial fibrosis and LGE. Previously, fibrosis was thought to result from tissue ischemia secondary to endothelial accumulation of glycosphingolipids in the microvasculature. In this study they compared CMR images and blood biomarkers of inflammation and myocardial damage (troponin) in FD, HCM, chronic myocardial infarction and healthy volunteers. FD patients had elevated T2 values in the LGE segments (particularly in the BILW) but also globally. This differed from the normal values found in controls and patients with myocardial infarction and was even higher than the T2 elevation seen in HCM patients. In addition, troponin was elevated in 40% of FD patients and only occurred when LGE was present. The strongest predictor of troponin elevation was T2 values in the BILW.

Augusto *et al*. [78], went one step further by demonstrating that T2 values are associated with elevation of other biomarkers such as and N-terminal pro-B-Type natriuretic peptide, changes in the electrocardiogram (ECG) and LV mechanical impairment (reduced global LS).

These results must be taking with caution as there is no histological validation to date or direct measures of the immune system. Nonetheless, previous studies have identified infiltration of lymphocytes and macrophages in the myocardium of FD patients who underwent endomyocardial biopsy [23]. In addition, patients with FD have significantly elevated plasma levels of inflammatory biomarkers such as tumor necrosis factor (TNF), TNF receptor 1 (TNFR1), TNF receptor 2 (TNFR2), interleukin-6 (IL-6), galectin-1 and galectin-3 compared to healthy controls [79, 80]. If confirmed in future studies, these findings could demonstrate a pivotal role for inflammation in FD pathogenesis and suggests that T2 and troponin levels could be new treatment and disease monitoring targets.

Possible mechanism of myocardial inflammation in FD are the accumulation of Gb3 and lyso-Gb3 that could act as antigens, activating the release of secondary mediators of injury and natural killer T-cells that lead to chronic inflammation an auto-immunity [81, 82].

### 4.3 Late Gadolinium Enhancement (Fibrosis)

The existence of focal fibrosis (irreversible) can be assessed by the presence and distribution of the LGE following the administration of contrast agents. The typical distribution of LGE in FD patients is in the mid myocardium layers of the BILW, the same region that has been reported to be the first to present mechanical dysfunction [83]. Why this region is affected in FD remains unknown. An ischemic etiology is unlikely since ischemic necrosis usually starts at the sub-endocardium. One hypothesis is increased local wall stress in the BILW since LV work load is highest in this region [55, 84]. The BILW is the most mobile of the basal segments and likely faces the most junctional stress transmitted from the fibrous skeleton into the myocardium [85]. Another explanation could be a higher sphingolipid deposition and inflammatory response in the aforementioned segments. Atypical patterns of LGE in the mid and apical LV have also been reported in the literature [85]. Curiously, patients with non-concentric LV hypertrophy (such as asymmetric septal hypertrophy that mimics HCM) had more total and atypical distribution of LGE [85].

## 5. Staging 

**Fig. 6.**
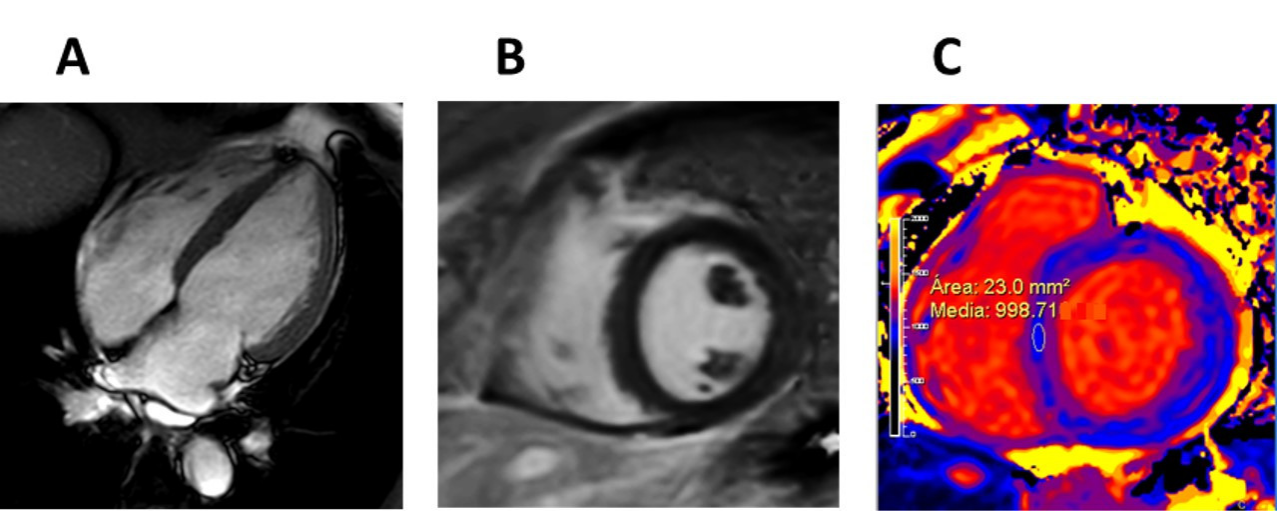
**Early stage Fabry disease**. (A) Four-chamber cardiac magnetic resonance (CMR) image of a patient with early stage Fabry disease showing no left ventricular hypertrophy. (B) Short-axis late gadolinium enhancement CMR image demonstrating no late gadolinium enhancement. (C) Short-axis CMR T1 colour map demonstrating reduced T1 signal.

**Fig. 7.**
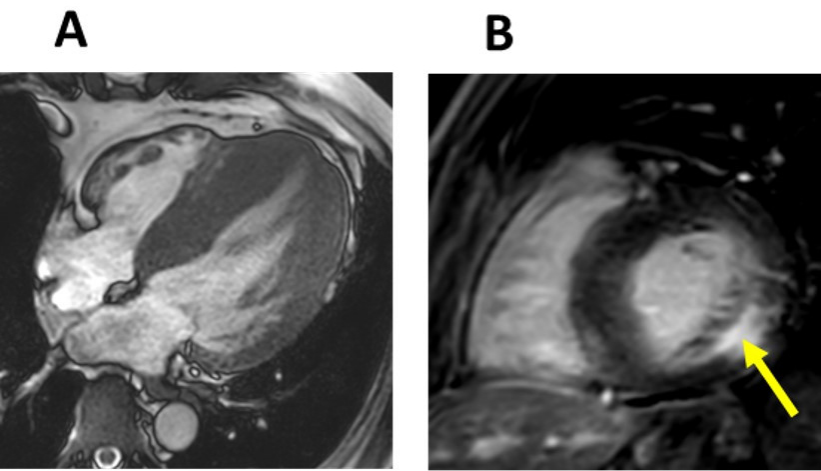
**Late stage Fabry disease**.(A) Four-chamber cardiac magnetic resonance (CMR) image of a patient with late stage Fabry disease showing severe concentric left ventricular hypertrophy. (B) Short-axis late gadolinium enhancement (LGE) CMR image demonstrating extensive LGE in the basal inferolateral wall (arrow).

Piecing together all of the previous findings, Nordin *et al*. [86] was able to construct a three-phase model of cardiac FD progression that subsequently expanded to include a fourth stage based on the findings of Augusto *et al*. [87]. The proposed phases are as follows: the microvascular, accumulation, inflammation and/or hypertrophy and the fibrosis and/or impairment phase; they are summarized in Table 2 and Figs. 6,7.

**Table 2.** **Stages of Fabry disease**.

		Microvascular/pre-accumulation	Accumulation	Hypertrophy/Inflammation	Fibrosis and/or impairment
Age		Childhood (Starts before birth)	Childhood/Adolescence	Adulthood	Adulthood
Pathophysiology		Lysosomal storage and activation of secondary pathways	Lysosomal storage and activation of secondary pathways	Hypertrophy and inflammation	Fibrosis
Symptoms		No cardiac symptoms (silent/subclinical detection)	No cardiac symptoms	Chest pain	HF-pEF → HF-rEF
		Autonomic and small fiber abnormalities (acroparesthesia, GI symptoms, impaired sweating) ↓ Heart rate variability in children		Arrhythmias	
				Reduced exercise capacity	
Echo-cardiogram	LVH	-	↑ LV/PM mass within normal limits	LVH	↑↑ LVH
	Strain	↓ GLS starting in the BILW	↓↓ GLS	↓↓ GLS	↓↓ GLS
		↓ GCS (loss of normal base-to-apex gradient)	↓ RS	↓ RS	↓↓ RS
	EF	Normal	Normal/↑	Normal/↑	↓
CMR	T1	Normal but falling	↓	↓↓	↓/Pseudo-normal
		Lower than healthy individuals			
	T2	Normal	Normal	↑ T2 Focal	↑↑ T2 Focal or global
			Occasionally ↑ (>females)		
	LGE	-	Occasional LGE (>females)	LGE in the BILW	Extensive LGE
	Other	↓ Myocardial blood flow			
ECG		Short P wave	Normal P wave time	Normal/ long P wave	Long P wave
		Low T wave amplitude	Normal T wave ratio	Elevated T wave ratio	Elevated T wave ratio
		Low T wave ratio			Increased QRS duration
Biomarkers		Novel biomarkers (metabolomics, proteomics, LVH pathways)	Gb3/LysoGb3	↑ Troponin	↑ NT-proBNP and Troponin (Fibrosis biomarkers)
Expected ERT efficacy		High	High	Intermediate	Low

AMVL, anterior mitral valve leaflet elongation; BILW, basal inferolateral wall; CMR, cardiac magnetic resonance; ECG, electrocardiogram; EF, ejection fraction; ERT, enzyme replacement therapy; Gb3, globotriaosylceramide; GCS, global circumferential strain; GI, gastrointestinal; GLS, global longitudinal strain; HF-pEF, heart failure with preserved ejection fraction; HF-rEF, heart failure with reduced ejection fraction; LGE, late gadolinium enhancement LVH, left ventricular hypertrophy; Lyso-Gb3, globotriaosylsphingosine; RS, radial strain; ST wave ratio = (T onset – T peak) / (T peak – T end).

### 5.1 Microvascular/Pre-Accumulation Stage 

Cardiac damage in FD begins early in life due to the accumulation of sphingolipids in practically all cardiac cell types and tissues (myocytes, endothelial cells, valvular fibroblasts and conduction tissue) [2]. The buildup of Gb3 triggers secondary processes such as the activation of neurohormonal pathways or the release of trophic factors such as sphingosine-1-phosphate (S1P) [34] that will lead to apoptosis and cellular hypertrophy. Direct accumulation of lyso-Gb3 can also activate these changes by itself [88].

Storage of Gb3 has been shown to start before birth [89] and progresses sub-clinically before we are able to detect it. Thus, a silent pre-accumulation stage exists and is characterized by microvascular dysfunction, impaired LV mechanics and altered ECG with normal T1 values.

Microvascular disfunction is an early marker of FD and could be the only sign of cardiac involvement in some patients. Early studies using PET and dipyridamole-induced maximal blood flow demonstrated that FD causes abnormal coronary function with low flow reserve [90]. A more recent study, comparing coronary microvasculature in 30 FD patients and 24 healthy controls, concluded that the alteration in coronary microvascular function seen in FD patients is not dependent on LVH or gender [91]. Similar results have been demonstrated using stress perfusion mapping with CMR that revealed reduced myocardial blood flow [92, 93]. Microvascular function did not improve after 12 months of ERT [94].

Concerning LV mechanics, Vijapuruapu *et al*. [48] showed that in FD patients without LVH, impairment in global LS was associated with a normal but decreasing value of native T1, suggesting that mechanical dysfunction occurs before evidence of sphingolipid deposition.

Patients usually do not present any cardiac symptoms in this phase. However, they could have symptoms related to autonomic and small fiber abnormalities (acroparesthesia, gastrointestinal disturbances, alterations in sweating …etc.). In fact, there are reports that suggest an alteration of parasympathetic cardiac stimulation evidenced by a reduction in heart rate variability in children with positive gene mutations for FD [95]. A hypothesis that could explain the early appearance of microvascular dysfunction and neurological damage is the greater susceptibility of neurons and endothelial cells to sphingolipid storage compared to myocytes.

Although no abnormalities are detected on a standard clinical and imaging assessment, some hearts already show electrocardiographic alterations in this phase. These include: a reduced T wave amplitude, shortening of P-wave duration reflecting accelerated intra-atrial conduction and shorter T onset – T peak time with a shorter T wave ratio ((T onset – T peak) / (T peak – T end)) that results in more symmetrical T waves.

### 5.2 Accumulation Stage 

In this phase, native T1 has decreased below normal values although LVH has not yet developed. Despite the absence of cardiac hypertrophy, minor increases of LV and PM mass within normal range can be seen in this stage [71]. In addition, LVEF can be slightly elevated revealing a hyperdynamic state.

### 5.3 Inflammation and/or Hypertrophy Stage

As the disease advances, LVH appears with a more severe and earlier presentation in men as previously mentioned. Thus, an elevated myocardial mass can be detected by CMR and echocardiography. With increasing LVH, impaired global LS develops proportionate to LV wall thickness.

There is evidence of inflammation starting in the BILW segment as suggested by the presence of LGE and elevated T2 values in this region without wall thinning. This phase is also associate with elevation of inflammatory biomarkers such as troponin.

Clinically, patients may suffer from chronic fatigue and less exercise capacity, but usually no overt heart failure (HF) symptoms are present [96]. Some functional impairment can be unmasked by exercise testing. Réant *et al*. [50] was the first study to demonstrate that echocardiographic parameters can predict functional status in FD patients. Reduced ${\mathrm{VO}}_{2}$ peak and increased ${\mathrm{VR/VCO}}_{2}$ slope (suggesting respiratory inefficiency and low cardiac output) were associated with global LS impairment and higher LV wall thickness, respectively.

As for the ECG changes, P-wave duration first pseudo-normalizes and finally prolongs in this phase due to extracellular expansion and left atrial remodeling that slow down intra-atrial conduction. Hence, P-wave duration in FD follows an interesting ‘biphasic’ pattern with disease progression. The same applies to the *T wave ratio* that is elevated in this stage.

### 5.4 Fibrosis and/or Impairment Stage

This is the most advanced cardiomyopathy phase and is characterized by replacement fibrosis recognized by the presence of LGE in CMR imaging. LGE extends beyond the BILW towards other basal and mid-myocardial segments of the LV and could cause wall thinning in the BILW.

Likewise, T1 tends to increase and pseudo-normalize. Possible mechanisms that could explain this fact are: the increase of myocardial hypertrophy versus storage component, increased fibrosis and myocardial inflammation [96, 97].

In the absence of therapy, all of these processes cause further deterioration in longitudinal and radial strain and a decline in LVEF resulting in HF signs and symptoms including elevated filling pressures and N-terminal pro-brain natriuretic peptide.

## 6. Sex Dimorphisms

**Fig. 8.**
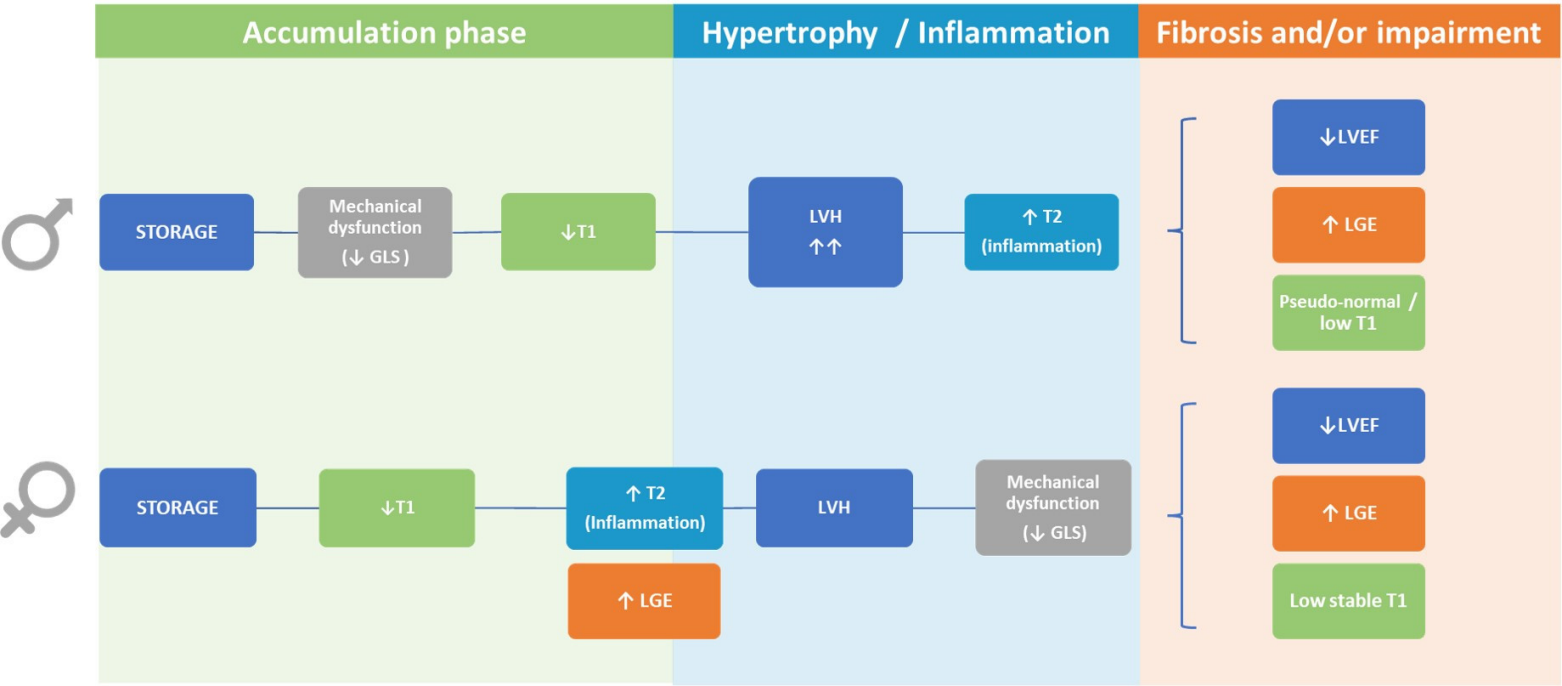
**Sex dimorphisms in Fabry disease**. Some female patients with FD can develop inflammation (elevated T2 values) and/or fibrosis (LGE) before the presence of LVH which is rare in males. On the other hand, mechanical dysfunction does not appear until the development of LVH in female patients. By contrast, male patients usually present mechanical dysfunction before LVH. Finally, in males, T1 values tend to pseudo-normalize in late stages due to a higher degree of hypertrophy and fibrosis compared to females in whom T1 values can remain low but stable. GLS, global longitudinal strain; LGE, late gadolinium enhancement; LVEF, left ventricular ejection fraction; LVH, left ventricular hypertrophy.

Overall, the four stages of cardiac involvement are common for both males and females. However, some sex dimorphisms have been previously proposed in FD (Fig. 8).

The first relates to the severity of hypertrophy which is far more extreme and has an earlier onset in men (even when indexed) suggesting faster storage [3]. One possible explanation is the different way men and women respond to storage: in men, Gb3 accumulation triggers “true LVH” due to myocyte hypertrophy rather than “storage LVH” caused by a balance of sphingolipid deposition and hypertrophy as seen in women [86]. This hypothesis is based on reports of the different relationship between LVH and T1 changes seen in both genders [48]. In women, T1 falls until LVH is present and then stabilizes. However, in men T1 can increase (to a more normal value) after the development of LVH. This increase in T1 could be due to myocyte hypertrophy that dilutes the T1 lowering caused by sphingolipid. This phenomenon has also been described in other cardiac diseases and may be due to differential expression of androgen and estrogen receptors and differences in the renin-angiotensin system, nitric oxide activity and norepinephrine release.

Secondly, mechanical dysfunction also appears to differ according to gender. Females tend to have preserved global LS until the presence of LVH, whereas males have impaired global LS with T1 lowering before the onset of LVH [48], suggesting a better tolerance to storage in women.

Lastly, inflammation and/or fibrosis can precede LVH in females [83, 98] but is rarely observed in males. Thus, T2 mapping and LGE are especially important for female patients as they might be the only way to detect a potential cardiomyopathy in women and could guide the initiation of specific treatment for FD in the absence of LVH. Further research is required to discern whether these different phenotypes (patients with LVH and inflammation/fibrosis versus patients without LVH but with inflammation/fibrosis) respond differently to treatment or have a different natural history.

## 7. Patient Selection for Treatment and Monitoring

Unlike other infiltrative cardiomyopathies, FD has the potential to stabilize with treatment. Nonetheless, treating all FD patients from diagnosis is not an option due to the financial burden it entails. Consequently, it is important to determine the optimal timing for intervention. The European Fabry working group [99] recommends initiation of ERT, independently of gender or phenotype (classical vs non-classical) in the presence of cardiac hypertrophy (myocardial wall thickness >12 mm) (class I recommendation) or signs of cardiac rhythm disturbances (class I). In males with classical FD that are 16-years of age or over, treatment could be initiated even in the absence of signs or symptoms of the disease (class IIB). However, the presence of myocardial fibrosis has been shown to negatively affect treatment outcomes. As a result, current guidelines do not recommend initiation of treatment in advanced cardiac disease with extensive fibrosis if no other organ is impaired [10].

**Fig. 9.**
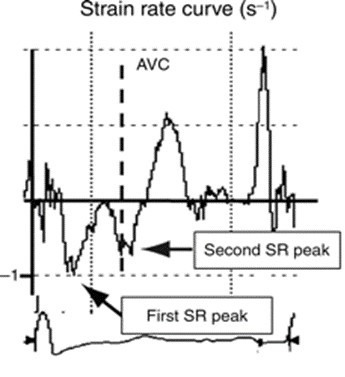
**Longitudinal strain rate curve over one heart cycle from a patient with aortic valve stenosis extracted from a segment with late gadolinium enhancement**. The typical ‘double peak sign’ with a first and a second strain rate peak is seen. *Note*. Reproduced from “A new echocardiographic approach for the detection of non-ischaemic fibrosis in hypertrophic myocardium by Weidemann *et al*. [100] European Heart Journal. 2007; 28: 3020–3026” by permission of Oxford University Press; AVC, aortic valve closure; SR, strain rate.

Therefore, identifying parameters of fibrosis is crucial to correctly select patients who can benefit from specific treatment. As mentioned before, fibrosis is mainly detected by CMR, but SR or speckle tracking could be useful for patients with contraindications or centers where this test is not available. Weidemann *et al*. [100] described that the myocardial segments that were affected by fibrosis showed a “double peak sign” in the SR tracings. This consists of a sharp first peak in early systole, followed by a rapid fall and a second strain rate peak during the isovolumetric relaxation period, corresponding to post-systolic shortening of the affected segment (Fig. 9, Ref. [100]). However, SR imaging has the disadvantages of being technically demanding, time consuming and difficult for post processing. Similarly, a systolic LS value of <12.5% in the BILW measured by speckle-tracking was strongly correlated with the presence of LGE in CMR. By contrast, values >16.5% makes fibrosis extremely unlikely [101].

Recently, a European panel of experts have published organ-specific therapeutic goals for patients with FD based on a systematic review and consensus opinion [102]. These include the prevention or stabilization of LVH and/or fibrosis, improvement of exercise capacity and quality of life in patients with HF symptoms, control of cardiovascular risk factors and treatment of arrhythmias such as atrial fibrillation or ventricular tachycardia.

Regarding the monitoring of the development and progression of FD cardiomyopathy, expert groups [5, 103] suggest that an ECG and echocardiogram should be performed in adult patients at diagnosis and annually regardless of symptoms, phenotype or whether they are receiving ERT. CMR is recommended in adults if there is evidence of clinical progression or regularly at an interval of more than two years. Adolescents should receive an echocardiogram every two years, whereas in pediatric patients a CMR is recommended at baseline and subsequently to monitor treatment efficacy or if disease progression is suspected.

## 8. Prognosis 

Patients with FD have a reduced life expectancy; death occurs at a mean age of 54 years in men and 62 years in women [4]. Since the availability of renal replacement therapy, the most common cause of death in FD patients has changed from renal to cardiovascular disease, mainly HF or arrhythmia [4, 104]. The most common adverse events in FD patients are also cardiological followed by renal, stroke and non-cardiac deaths [105].

Various imaging findings have been identified as predictors of poor prognosis in FD. For example, the degree and presence of LVH has been associated with reduced event-free survival [49] and was one of the strongest predictors of major cardiovascular events in the Fabry registry, including myocardial infarction, HF and sudden cardiac death [105]. It is also correlated with a greater risk of arrhythmia, valvular disease and increased intima-media thickness of the common carotid artery in this population [106, 107]. Increased trabecular and PM volume have also been associated with overall arrhythmia, atrial fibrillation and ventricular tachycardia [85].

Conversely, there is a lack of data regarding the prognostic value of LV function in FD patients. In order to shed some light on the issue, Spinelli *et al*. [108] evaluated the predictive value of various parameters of LV function in FD patients with a normal LVEF. These parameters included: LV diastolic function indices, global LS and novel measurements of LV function such as myocardial work (MW). Their findings suggest that LV function impairment (both systolic and diastolic) is associated with adverse events in FD. Moreover, global LS and MW were independent predictors of adverse cardiac outcomes with MW showing the highest sensitivity and specificity for predicting adverse outcomes as analyzed by ROC curve analysis. However, MW did not improve the predictive value of a model including clinical data, LV mass, LV diastolic function and global LS.

As for CMR parameters, T1 mapping could be useful to track disease progression in early stages. A study of 44 Fabry patients without LVH, found that low T1 was a risk factor for clinical worsening at 12-month follow-up [93]. However, T1 fails to predict advanced stages of the disease due to its pseudo-normalization. In contrast, T2 increases with disease progression and has shown to have a prognostic value. Augusto *et al*. [78] demonstrated that increased T2 values were associated with clinical worsening after one year in FD patients. Likewise, various studies have found an association between the presence and extent of LGE and a greater risk of adverse cardiac events in FD, particularly ventricular tachycardia. These findings have a biological explanation since fibrosis is a known substrate for arrythmia [109, 110]. In addition, Réant *et al*. [50] found a significant correlation between cardiopulmonary exercise parameters such as ${\mathrm{VE/VCO}}_{2}$ slope and the occurrence of atrial fibrillation and stroke, the most frequent complications suffered by FD patients. LV wall thickness, basal LS and T1 values also predicted adverse events in this study [50].

In summary, LV wall thickness, LV function (diastolic indices, basal and global LS and MW), CMR metrics (T1, T2 values and LGE) and cardiometabolic parameters (${\mathrm{VE/VCO}}_{2}$ slope) have all shown to be independent predictors of worse outcomes in FD.

## 9. Conclusions

The current review emphasizes the importance of multimodal imaging for the management of patients with FD. The echocardiography continues to be the technique of choice for initial evaluation and follow-up of these patients with LVH as the hallmark feature of FD cardiomyopathy. However, CMR is gaining importance in recent years as it provides more accurate and reproducible measurements of cardiac volume, function and mass. Advances in tissue characterization by CMR have led to a more accurate model of disease progression and staging.

Novel imaging techniques have emerged as a possible solution to some of the main concerns of FD patients. These problems include the diagnostic delay and the dilemma as to when is the optimal time to initiate disease-specific treatment and what is the best biomarker to monitor treatment response. Speckle-tracking, TDI and CMR can aid subclinical detection of FD before irreversible fibrosis develops. Future studies are needed to determine if initiating specific treatment for FD when LV wall thickness is normal but subclinical parameters are impaired improves clinical outcomes. Finally, the discovery of the pivotal role of inflammation in FD opens the door to the development of new therapies that target inflammation and highlights the use of T2 or troponin as biomarkers to monitor the response to treatment. All of these advances will ultimately contribute to improve the outcomes of patients suffering from this rare disease.

## Abbreviations

AD, autosomal dominant; α-Gal A, alfa-galactosidase A; AR, autosomal recessive; AMVL, anterior mitral valve leaflet elongation; AV, atrioventricular; BILW, basal inferolateral wall; CS, circumferential strain; CMR, cardiac magnetic resonance; CPEO, chronic progressive external ophthalmoplegia; CPK, creatine phosphokinase; ECG, electrocardiogram; ECV, extracellular volume; EF, ejection fraction; ERT, enzyme replacement therapy; FD, Fabry disease; FOS, Fabry Outcome Survey; FXN, frataxin; Gb3, globotriaosylceramide; GCS, global circumferential strain; GI, gastrointestinal; GLS, global longitudinal strain; HCM, hypertrophic cardiomyopathy; HF, heart failure; HF-pEF, heart failure with preserved ejection fraction; HF-rEF, heart failure with reduced ejection fraction; IL-6, interleukin-6; KSS, Kearns-Sayre syndrome; LAMP2, Lysosome-associated membrane protein 2; LGE, late gadolinium enhancement; LS, longitudinal strain; LVEF, Left ventricular ejection fraction; LVH, left ventricular hypertrophy; LVOTO, left ventricular outflow tract obstruction; Lyso-Gb3, globotriaosylsphingosine; MELAS, mitochondrial encephalomyopathy; MV, mitral valve; MW, myocardial work; NT-proBNP, N-terminal pro-brain natriuretic peptide; PET, positron emission tomography; PM, papillary muscle; RLS, regional longitudinal strain; RV, right ventricle; RVH, right ventricular hypertrophy; SAM, systolic anterior motion; SR, strain rate; STIR-T2, T2-weighted short-tau inversion recovery; S1P, sphingosine-1-phosphate; TDI, Tissue Doppler Imaging; TNF, tumor necrosis factor; TNFR, tumor necrosis factor receptor; TTR, transthyretin; ${\mathrm{VE/VCO}}_{2}$slope, relationship between minute ventilation and carbon dioxide production; ${\mathrm{VCO}}_{2}$, carbon dioxide production; ${\mathrm{VO}}_{2}$, oxygen consumption; WPW, Wolff-Parkinson-White; WT-TTR, wild type transthyretin amyloidosis.
